# PCR and Culture Analysis of *Streptococcus pneumoniae* Nasopharyngeal Carriage in Healthy Children

**DOI:** 10.3390/microorganisms9102116

**Published:** 2021-10-08

**Authors:** Leah J. Ricketson, Ravinder Lidder, Robyn Thorington, Irene Martin, Otto G. Vanderkooi, Manish Sadarangani, James D. Kellner

**Affiliations:** 1 Department of Pediatrics, Cumming School of Medicine, University of Calgary, Calgary, AB T2N 1N4, Canada; ljricket@ucalgary.ca (L.J.R.); Otto.Vanderkooi@albertahealthservices.ca (O.G.V.); 2 National Microbiology Laboratory, Public Health Agency of Canada, Winnipeg, MB R3E 3R2, Canada; Ravinder.lidder@phac-aspc.gc.ca (R.L.); robyn.thorington@phac-aspc.gc.ca (R.T.); irene.martin@phac-aspc.gc.ca (I.M.); 3 Alberta Children’s Hospital Research Institute, Calgary, AB T3B 6A8, Canada; 4 Vaccine Evaluation Center, BC Children’s Hospital, Vancouver, BC V6H 3N1, Canada; msadarangani@bcchr.ubc.ca; 5 Department of Pediatrics, University of British Columbia, Vancouver, BC V6T 1Z4, Canada

**Keywords:** *Streptococcus pneumoniae*, nasopharyngeal carriage, serotype, polymerase chain reaction

## Abstract

Invasive *Streptococcus pneumoniae* disease is preceded by asymptomatic nasopharyngeal carriage. Measuring carriage in healthy populations provides data on what serotypes are present in communities, which is of interest in the era of polyvalent pneumococcal conjugate vaccines. Nasopharyngeal swabs from a survey of 682 and 800 healthy children in 2016 and 2018, respectively, were analyzed by culture and Quellung reaction to determine rates of carriage and serotypes. All swabs from 2016 and 300 randomly selected swabs from 2018 were then analyzed using real-time semi-quantitative PCR (qPCR) to detect *S. pneumoniae* gene targets *lytA, piaA*, and SP2020 and determine serotype. There were 71 (10.4%) and 68 (8.5%) culture positive samples in 2016 and 2018, respectively. All of these were also positive by qPCR except one that was equivocal. In total, 46.0% of 2016 swabs were positive by qPCR. In 2018, results from the selected sample extrapolated to the complete sample showed 49.0% positive by qPCR. PCV13 serotypes were detected in 29.3% and 21.7% of *S. pneumoniae* qPCR positive samples from 2016 and 2018, respectively; compared with only 8.4% and 6.0% PCV13 serotypes detected by Quellung reaction in culture positive samples. Compared with culture, qPCR detected *S. pneumoniae* more frequently. Further, qPCR serotyping detected PCV13 serotypes in a larger proportion of samples than culture and Quellung reaction did, showing that, despite established universal childhood PCV13 immunization, vaccine serotypes can still be detected in a large proportion of young children.

## 1. Introduction

*Streptococcus pneumoniae* (pneumococcus) is an opportunistic pathogen because it colonizes asymptomatically, with carriage being a prerequisite for disease [1,2]. Carriage, especially in children, can act as an important reservoir that facilitates horizontal spread of *S. pneumoniae* throughout a community [1].

The seven-valent pneumococcal conjugate vaccine (PCV7) targets seven serotypes (4, 6B, 9V, 14, 18C, 19F, and 23F) and was introduced in the province of Alberta, Canada in 2002 with three doses given before a year of age and a single booster after one year (3 + 1 schedule) [3]. In 2010, Alberta introduced the thirteen-valent pneumococcal conjugate vaccine (PCV13) with a 2 + 1 schedule, which added serotypes 1, 3, 5, 6A, 7F, and 19A to the serotypes already included in the PCV7 vaccine [3]. There is a need for continual monitoring of the carriage of *S. pneumoniae* in children to understand herd immunity and serotype replacement, as well as invasive and non-invasive disease by vaccine serotypes [4]. In the city of Calgary, Alberta, after the introduction of PCVs, we have noted not only a decline in carriage of vaccine serotypes, but a decline in overall carriage of *S. pneumoniae* as identified by standard culture [5]. 

Most surveillance studies use conventional culture methods and Quellung reaction serotyping [6] for detection of *S.* *pneumoniae* from patients with non-invasive or invasive disease or nasopharyngeal carriage. However, it is recognized that some samples may be falsely reported as negative because the bacteria cannot be grown in culture [7]. This may be an issue in particular for nasopharyngeal samples where the bacterial load on a nasopharyngeal swab may be very low [8]. Polymerase chain reaction (PCR) can be used to detect the presence of pneumococcal DNA without relying on the growth of live cells in culture. This means that PCR detects a higher number of positive samples than culture methods [7,9]. Furthermore, the increased sensitivity of PCR can lead to the concurrent detection of multiple serotypes in a single person. Although detection of *S. pneumoniae* DNA by PCR cannot distinguish live, transmissible bacteria from non-living, non-transmissible bacterial remnants, the increased sensitivity to detect evidence of bacteria that are, or were recently, viable and transmissible, may yield more useful epidemiologic data than culture studies can. This is useful for studies evaluating whether PCV-related serotypes continue to circulate in communities with universal vaccine programs. Studies that compared the sensitivity of conventional PCR vs real-time PCR (qPCR) for the detection of *S. pneumoniae* serotypes have shown that qPCR assays are more sensitive to the presence of *S. pneumoniae* [10,11].

We hypothesized that in the era of routine PCV use, children may carry a lower quantity of *S. pneumoniae* in their respiratory tracts that will be better identified using molecular methods rather than conventional culture. The purpose of this study was to investigate and understand how often *S. pneumoniae* may be detected by qPCR but not by conventional culture methods. We also compared serotyping results by Quellung reaction and qPCR. 

## 2. Materials and Methods

### 2.1. Original Surveys to Collect Samples and Identify S. pneumoniae by Culture and Quellung Serotyping

The Calgary Area *Streptococcus pneumoniae* Epidemiology Research (CASPER) team conducted 13 point-prevalence surveys periodically from 2003–2018 in healthy Calgary children to determine overall and serotype-specific trends in pneumococcal nasopharyngeal carriage, identified by culture and Quellung serotyping. Results from these surveys have been previously published [5,12]. Nasopharyngeal samples were collected according to the methods reported previously [5,12]. Samples in this study were collected between 11 April 2016, and 2 June 2016, and between 7 May 2018, and 22 June 2018.

All subjects provided informed consent at the time of swab collection. The study was approved by the University of Calgary Conjoint Health Research Ethics Board (Ethics ID REB15-0571).

At the time of each survey, nasopharyngeal swabs were transported at an ambient temperature to the Alberta Precision Laboratories (formerly Calgary Laboratory Services) where *S. pneumoniae* was identified through standard culture methods using colony morphology, alpha hemolysis, optochin sensitivity, and bile solubility tests [13,14]. After being swabbed on culture plates, all nasopharyngeal swabs collected in 2016 and 2018 were frozen at −85 °C for future use. In 2016, 71/682 samples (10.4%) were positive by culture for *S. pneumoniae*, and in 2018, 68/800 samples (8.5%) were positive by culture. 

At the time of each survey, *S. pneumoniae* isolates identified by culture were frozen and sent to the National Microbiology Laboratory (NML) for serotyping using the Quellung reaction with antisera obtained from SSI Diagnostica; Statens Serum Institute (Copenhagen, Denmark). More detailed methods are reported elsewhere [5,12].

### 2.2. Identification of S. pneumoniae and Serotypes by qPCR

For the current study, in 2019, all frozen nasopharyngeal samples (*n* = 682) from the 2016 carriage survey were sent to the NML for qPCR analysis. Later, in early 2020, a subset of 300 of the 800 nasopharyngeal samples from 2018 were also sent to NML. The 2018 subset included all nasopharyngeal samples that were positive by culture methods (*n* = 68) and a random selection of culture negative samples (*n* = 232). Not all samples collected in 2018 were sent for qPCR reasons due to study budget limitations. Investigators at NML were blinded about the culture results from all swabs until after testing was completed. 

Target DNA was isolated using the Roche MagNA Pure 96 (Roche Diagnostics, Basel, Switzerland) automated extraction platform by processing 500 µL of swab transport media with the MagNA Pure 96 DNA and Viral NA Large Volume Kit (Roche Diagnostics, Basel, Switzerland) producing a 50 µL total nucleic acid eluate (as per manufacturer instruction). Extracted samples were verified for DNA isolation and integrity by PCR amplification of the Human Beta-globin endogenous gene target (internal control) [15].

*Streptococcus pneumoniae* qPCR detection assays were performed on the extracted DNA using the Qiagen QuantiNova SYBR Green PCR kit (Qiagen, Germantown, MA, USA) with 0.7 µM primer concentration. Samples were screened for *S. pneumoniae* using qPCR targets *lytA* (autolysin gene) [16] and *piaA* (a lipoprotein component of the pneumococcal ABC transporter gene) [17]. An additional target, SP2020 (putative transcriptional regulator gene) [18], was used if the first two targets showed one negative result and one positive. A positive result of any two targets was considered positive for S. *pneumoniae* with no priority assigned to any particular target. An equivocal result was a single positive target. The qPCR detection primers pairings have sensitivity and specificity between 95% and 100% [16,17,18]. All positive and equivocal results continued to serotype. Equivocal results that could be serotyped were considered to be positive, while not typable samples with a single positive qPCR target remained classified as equivocal (Table 1). Detection of *S. pneumoniae* by qPCR was performed on the ThermoFisher Scientific Applied Biosystems QuantStudio 5 Real-time PCR (ThermoFisher Scientific, Waltham, MA, USA) instrument and the cycling conditions were as follows: 95 °C hold 2 min, 95 °C 10 s and 60 °C 30 s for 40 cycles, 60 °C for 2 min final extension, melt curve analysis 70 °C to 95 °C at 0.1 °C per second with optics. 

Serotypes were determined using the Center for Disease Control’s real-time PCR detection of 37 of 101 known pneumococcal serotypes (including all PCV13 serotypes and most other common serotypes) [10,19] using ThermoFisher Scientific TaqPath ProAmp PCR Master (ThermoFisher Scientific, Waltham, MA, USA) mix with primer and probe concentrations at 0.75 µM final and 0.5 µM final concentration, respectively. Table S1 details the serogroups and serotypes included in this assay, along with the fluorescent dye label used for each probe. The qPCR for serotyping was performed on ThermoFisher Scientific Applied Biosystems QuantStudio 5 Real-time PCR instrument following cycling conditions: 60 °C pre-read 30 s (optics), 95 °C polymerase activation 5 min, 95 °C 15 s and 60 °C 1 min (optics) for 45 cycles, 60 °C 1-min post-read (optics). These conditions were optimized by NML throughout the process. Results were compared to conventional culture and Quellung serotyping for both 2016 and 2018 collections. A cycle threshold (CT) of <40 was used to indicate a positive serotype reaction [11,20,21]. Results that could not be serotyped by Quellung reaction are referred to as non-typeable, whereas samples that did not match one of the 37 serotypes detected by qPCR are referred to as “not typed”.

### 2.3. Statistical Analysis

**Figure 1:**
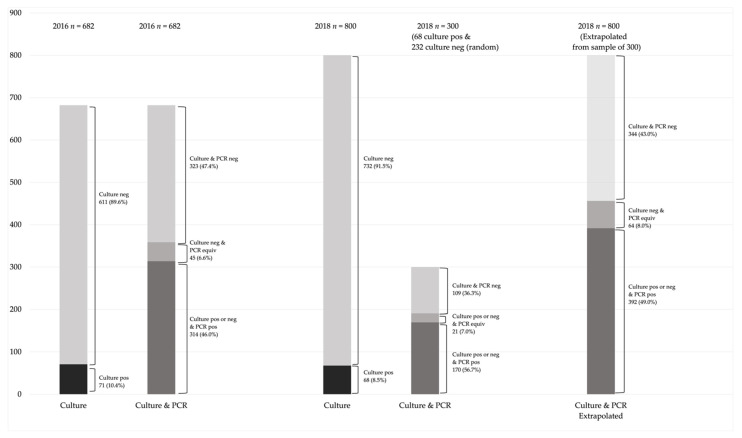
Culture and PCR results for 2016 and 2018, positive, negative, and equivocal.

This study was largely a descriptive analysis, reporting on proportions of negative and positive results by culture and qPCR and proportions of serotypes found by Quellung and qPCR. For the 2018 survey, where only a random sample of 232 culture-negative swabs were assessed by qPCR, we applied the proportion of negative, equivocal, and positive PCR results to the entire sample of culture-negative results (*n* = 732 culture-negative). The results from this extrapolation were then added to the results for the 68 culture-positive swabs (1 equivocal, 67 positives by qPCR) to determine final numbers for Figure 1 applied to the full 2018 sample (*n* = 800).

We used a two-sample test of proportions to compare the proportion of samples from each survey that were positive for *S. pneumoniae* identified by culture and PCR in order to look for statistical significance. We also compared the proportion of serotypes (vaccine related serotypes or not) identified by Quellung and qPCR. PCV13-related serotypes that could not be distinguished from the vaccine serotype by qPCR analysis (e.g., 18C/F/B/A) were grouped with PCV13 serotypes for Table 2 and Table 3. Serotypes 6C/D were also included as PCV13-related serotypes for Table 2 and Table 3 due to evidence of cross-reactivity for these serotypes with PCV13 serotypes 6A/B.

## 3. Results

### 3.1. Analysis of 2016 Samples

The 2016 survey included nasopharyngeal swab samples from 682 children. All 71 culture-positive swabs were also qPCR positive. In addition, 243/611 culture-negative swabs were qPCR positive and 45/611 of culture-negative swabs were equivocal by qPCR (Figure 1). Thus, a total of 314/682 total samples (46.0%) were qPCR positive and a 45/682 total samples (6.6%) were qPCR equivocal. There was a significantly higher number positive by qPCR than by culture (46.0% vs 10.4%, *p* < 0.001).

Real-time PCR serotyping results from 2016 are shown in Table 2. By Quellung reaction, 6 (8.4%) of the 71 culture-positive samples had PCV13-related serotypes, while 64 (90.1%) were non-vaccine serotypes (NVT) and 1 (1.4%) was non-typeable. From the 314 qPCR positive samples, a single serotype was identified by qPCR for 135 (43.0%) samples, while 2 or more serotypes were identified in 37 (11.8%) samples and 142 (45.2%) could not be serotyped due to the limited number of serotypes in the qPCR assay. In total, 92/314 (29.3%) PCR positive samples had 1 or more PCV13 serotypes compared to 8.4% culture positive samples that had PCV13 serotypes by Quellung reaction (*p* < 0.001).

The serotype identified by Quellung was not identified by qPCR in 14 of 71 culture positive samples. In one sample there was a mismatch with serotype 19A identified by Quellung and qPCR could not identify a serotype. For the other 13, the serotype found by Quellung was a serotype that could not be detected by qPCR as it was not one of the 37 serotypes included in the assay.

### 3.2. Analysis of 2018 Samples

The 2018 survey included 800 children. All 68 culture-positive nasopharyngeal swab samples were also qPCR positive except one sample that yielded an equivocal qPCR result. A random selection of 232/732 culture negative swabs were also selected for qPCR analysis, for a total of 300 samples. The qPCR results from the culture-negative swabs (*n* = 232) were extrapolated to apply to the entire sample of culture-negative swabs (*n* = 732). There were 103/232 culture-negative swabs that were qPCR positive and 20/232 culture-negative swabs that were equivocal by qPCR. The extrapolated total of culture-negative swabs that were estimated to be qPCR positive for *S. pneumoniae,* combined with the 68 culture-positive, was 392/800 (49.0%) and 64/800 (8.0%) with equivocal results (Figure 1). There was a significantly higher number estimated to be positive by qPCR than by culture (49.0% vs. 8.5%, *p* < 0.001).

Serotyping results from 2018 are shown in Table 3. By Quellung reaction, 4 (6.0%) of the culture-positive samples had PCV13-related serotypes, while 63 (94.0%) were NVT, 0 were non-typeable and 1 could not be serotyped as it was non-viable after storage. From the 170 PCR positive samples for 2018, a single serotype was identified by qPCR for 74 (43.5%) samples, while 2 or more serotypes were identified for 23 (13.5%) and 73 (42.9%) could not be serotyped due to limitation of the assay. In total, 37/170 (21.7%) samples had 1 or more PCV13 serotypes, which was significantly more than found by Quellung (*p* < 0.001).

The serotype identified by Quellung was not identified by qPCR for 17 of 68 culture positive samples. As with the 2016 samples, the serotype found by culture was a serotype that could not be detected by qPCR as it was not one of the 37 serotypes included in the assay.

Table S2 shows the isolates with Quellung reaction identified serotypes which are not included in the molecular assay, but also had additional qPCR detected serotypes for both years. For 30 of these 31 samples, 2 or more serotypes were identified by the combination of Quellung and PCR, which would suggest co-carriage. Quellung was repeated on all of these samples, confirming the original serotype results.

## 4. Discussion

In the era of established PCV use, it is important to understand how well vaccination is controlling disease and asymptomatic nasopharyngeal carriage caused by vaccine serotypes, as well as the overall level of *S. pneumoniae* carriage. In the current study, we found that qPCR detected *S. pneumoniae* more frequently than conventional culture in nasopharyngeal samples obtained from healthy children, long after the introduction of PCVs in Canada and after we started to observe reduced overall *S. pneumoniae* carriage using standard culture methods [5]. Further, qPCR serotyping detected PCV13 serotypes in a larger proportion of *S. pneumoniae*-positive samples than did Quellung serotyping. It is important to note that the nasopharyngeal samples collected in our surveys were from healthy children and collected for research and surveillance purposes only. Thus, it was beyond the scope of our study to determine whether each strain of *S. pneumoniae* that was identified by culture or qPCR was transmissible. However, our findings do suggest that PCV13 serotype strains of *S. pneumoniae* are still commonly identified in our population by qPCR, if not by culture, from asymptomatic carriage samples. Concurrent studies of clinical samples from cases of invasive pneumococcal disease (IPD) in Canada have identified that PCV13 serotypes are causing 26% of all cases of IPD in children and 31% of IPD in all ages during the same time period as these surveys [22,23]. Thus, the current findings, taken with previously reported findings on IPD serotypes, indicate that although universal use of childhood PCVs has reduced vaccine serotype invasive disease, vaccine serotypes have not been eliminated [23].

Other studies have compared the quantitative load of *S. pneumoniae* in nasopharyngeal samples collected from sick patients diagnosed with bacteremic pneumococcal infections. For example, Messaoudi et al. found that the nasopharyngeal concentration of serotypes also found in clinical blood cultures was 3 log-fold higher than serotypes found only in the nasopharynx and not in blood [24].

The CASPER team has conducted 13 point-prevalence studies measuring nasopharyngeal carriage, using conventional culture methods in convenience samples of healthy young children during routine immunization visits at public health centers in Calgary since 2003 [5,12]. In addition to sharp declines in vaccine-serotype carriage, overall *S. pneumoniae* carriage declined following widespread use of PCVs. In Calgary, PCV7 was introduced in 2002 and PCV13 was introduced in 2010. Prior to 2010, the overall carriage rate was 19.9%, but in 2010–2012, nasopharyngeal carriage declined to 13.2% [5]. In 2016 and 2018, the overall carriage rates were further reduced to 10.6% and 8.6%, respectively. The current study compares and bridges culture and qPCR results from the two most recent surveys, which was enabled by freezing and retaining nasopharyngeal swabs first used for culture and later for qPCR. Swabs collected from prior surveys were not retained and so no further comparisons can be made to determine if the differences in detection of *S. pneumoniae* by culture and qPCR would have been as large in previous years in which culture detection was higher.

While numerous other studies of *S. pneumoniae* carriage in children in the PCV era have reported no change in overall carriage levels (due to replacement of vaccine serotype strains with non-vaccine serotype strains), there is also evidence from clinical trials and other observational studies of reduced overall carriage similar to what we have reported [25,26].

The targets used for PCR detection of *S. pneumoniae* have evolved over time [27,28]. The current method of choice for detection of *S. pneumoniae* by qPCR is *lytA* (autolysin gene) [16]. The *lytA* primer and probe used in this study were designed by da Gloria Carvalho et al. to accurately differentiate *S. pneumoniae* from other closely related *Streptococcus mitis* group species [16]. The second detection target used in this study is the *piaA* (a lipoprotein component of the pneumococcal ABC transporter gene), which is used to increase the specificity of pneumococcal identification, although this target has been reported as being absent from some non-encapsulated pneumococci [17]. A recent report described an additional target, SP2020 (putative transcriptional regulator gene) [18], used along with *lytA* as a powerful strategy for the identification of *S. pneumoniae*. In this study, all three targets (*lytA, piaA* and SP2020) were used, but there was no priority assigned to any targets and positive or equivocal categorization was based on any combination of targets as well as serotyping results (Table 1). Given that each of the primers used have very high but not 100% sensitivity to detect *S. pneumoniae*, it is expected that by chance, only a single primer will be positive in some samples, leading to the equivocal designation if no serotype was identified.

Samples from each survey were analyzed by qPCR for *S. pneumoniae* detection and serotyping on separate occasions at the NML and the methods were refined on each occasion. The Centers for Disease Control’s *S. pneumoniae* molecular serotyping assay [10] was adapted and optimized. The assay detects 37 serogroups/serotypes, which are either included in the PCV13 vaccine or are otherwise common serotypes causing pneumococcal disease [10]. As a result, current qPCR serotyping assays are not as complete as Quellung reaction serotyping, since not all known serotypes are identified. Samples that are non-typeable with the Quellung reaction indicates that a cultured *S. pneumoniae* strain is not one of 101 known serotypes [19,29,30]. Conversely, samples that are not typed with qPCR serotyping would be expected to most often still have a known serotype that is not one of the 37 identified by the qPCR assay. Further, qPCR serotyping in some cases only detects groups of serotypes e.g., serotypes 18CFBA, whereas Quellung reaction can distinguish between each of the individual serotypes: 18C/18F/18B/18A.

In this study, when qPCR serotyping results detected a serotype group which included a PCV13 serotype e.g., 18C/18F/18B/18A (of which 18C is PCV13 serotype), we designated it as a PCV13 serotype. Thus, there may be an overestimate of the proportion of samples with vaccine serotypes if the actual serotype for these samples was non-PCV13. However, this only applied to a small number of samples with limited serotypes (6A/B/C/D, 9V/A, and 18C/F/B/A) and the non-PCV13 serotype(s) in these groups are rare. 

There was just one discrepancy in the serotypes determined by the Quellung reaction and qPCR, with a sample from 2016. The sample was positive by culture and qPCR but not typed by qPCR serotyping although the serotype found by Quellung (19A) was included in the qPCR assay, and therefore should have been detectable by qPCR. The Quellung was repeated for this sample confirming the 19A result. It is possible that in the clinical sample, the concentration of the DNA was too low to be detected by the qPCR. In one study that compared PCR and Quellung serotyping, 19A was frequently involved in discordant results [31]. When Quellung reactions were repeated, the Quellung result often changed to match the PCR result, while the PCR results were the same on repeat analysis, suggesting errors with the Quellung reaction may be more common [31]. Thirty other samples in this study had a different serotype found by qPCR than by Quellung, but the Quellung serotype was a serotype not included in the qPCR assay; therefore, it could not have been identified by qPCR. We did not consider these to be discrepant serotypes, but rather evidence of co-carriage by the serotype identified by Quellung and the one identified by qPCR.

PCV13-related serotypes were found more often by qPCR than by culture, suggesting vaccination may have reduced the quantitative load of bacteria with these serotypes, but not eliminated them. Of particular interest was the presence of serotype 4 in 3.5% of the samples by qPCR but 0% in culture-positive samples. There has been a recent outbreak of serotype 4 among adults in Calgary, especially homeless persons [32]. The findings from the current study suggest that children may still be an important reservoir for serotype 4. Similarly, other PCV13 serotypes, including 3 and 5, and to a lesser degree, serotypes 7F and 1, were not identified by culture/Quellung analysis but were frequently found by qPCR. Serotype 3 and 7F also continue to cause high levels of disease throughout Canada despite widespread vaccination [23]. In contrast, serotype 19A, which is also a vaccine type that continues to cause disease in Canada, was not commonly found in carriage by PCR or by culture (Table S3).

Serotypes 11A, 22F, 15A, 16F, and 23A were common in carriage but are not included in current PCVs, although 11A and 22F are included in the 23-valent pneumococcal polysaccharide vaccine [33]. All of these serotypes are among the more common serotypes causing disease in Canada between 2014 and 2018 [23]. Serotype 33F was less common but also present by qPCR. Serotypes 22F and 33F are included in a new 15-valent PCV [34] and they are also included along with serotypes 8, 10A, 11A, 12F, and 15B in a new 20-valent PCV [35].

## 5. Conclusions

Compared with culture, qPCR detected *S. pneumoniae* far more frequently in nasopharyngeal samples obtained from healthy children. In addition, qPCR serotyping detected PCV13 serotypes in a higher proportion of samples with *S. pneumoniae* than Quellung reaction serotyping did, showing that, despite established universal childhood PCV13 immunization, asymptomatic carriage of vaccine serotypes has not declined to the very low levels indicated by studies using culture and Quellung reaction serotyping only. Future studies of *S. pneumoniae* carriage may preferentially use PCR detection and serotyping in place of culture and Quellung techniques, particularly if more serotypes can be added to the existing PCR assays.

## Figures and Tables

**Table 1:** Assay interpretation for *S. pneumoniae* Detection with *lytA*, *piaA and* SP2020, Followed by Serotyping.

lytA	piaA	SP2020	Serotyping	Result
Pos ^a^	Pos	N/D	Serotyped or Not typed	Positive
Pos	Neg	Pos	Serotyped or Not typed	Positive
Neg	Pos	Pos	Serotyped or Not typed	Positive
Pos	Neg	Neg	Serotyped	Positive
Neg	Pos	Neg	Serotyped	Positive
Pos	Neg	Neg	Not Typed	Equivocal
Neg	Pos	Neg	Not Typed	Equivocal
Neg	Neg	N/D	N/D	Negative

^a^ Pos = Positive, Neg = Negative, N/D = Not done.

**Table 2:** Real-time PCR Identification of *S. pneumoniae* Serotypes from 314 Nasopharyngeal Samples that were qPCR Positive for *S. pneumoniae*, 2016 Survey.

	qPCR Results for All Samples *n* (%)	qPCR Results for Culture Positive Samples *n* (%)	qPCR Results for Culture Negative Samples *n* (%)
**PCV13/Related Serotype Carriage ^a^**	61 (19.4)	9 (12.7)	52 (21.4)
**NVT Carriage**	80 (25.5)	21 (29.6)	59 (24.3)
**Multi-Carriage by NVT and PCV13-Serotypes**	31 (9.9)	6 (8.4)	25 (10.3)
**Not Typed ^b^**	142 (45.2)	35 (49.3)	107 (44.0)
**Total Positive by qPCR**	314 (100.0)	71 (100.0)	243 (100.0)

^a^ Due to qPCR serotyping not distinguishing between related serotype groups (e.g., 18ABCD) all serotypes related to a PCV13 serotype that could not be distinguished or have evidence of cross reactivity (e.g., 6C/D) were grouped with the PCV13 serotypes. ^b^ The not typed could not be serotyped due to not matching one of the 37 serotypes detected by the qPCR assay.

**Table 3:** Real-time PCR Identification of *S. pneumoniae* Serotypes from 170 Nasopharyngeal Samples that were qPCR Positive for *S. pneumoniae*, 2018 Survey.

	qPCR Results for All Samples *n* (%)	qPCR Results for Culture Positive Samples *n* (%)	qPCR Results for Culture Negative Samples *n* (%)
**PCV13/Related Serotype Carriage ^a^**	29 (17.1)	11 (16.4)	18 (17.5)
**NVT Carriage**	60 (35.3)	29 (43.3)	31 (30.1)
**Multi-Carriage by NVT and PCV13-Serotype**	8 (4.7)	2 (3.0)	6 (5.8)
**Not Typed ^b^**	73 (42.9)	25 (37.3)	48 (46.6)
**Total Positive by qPCR**	170 (100.0)	67 (100.0)	103 (100.0)

^a^ Due to qPCR serotyping not distinguishing between related serotype groups (e.g., 18ABCD) all serotypes related to a PCV13 serotype that could not be distinguished or have evidence of cross reactivity (e.g., 6C/D) were grouped with the PCV13 serotypes. ^b^ The not typed could not be serotyped due to not matching one of the 37 serotypes detected by the qPCR assay.

## Data Availability

The dataset for this study will be available in the University of Calgary PRISM Repository.

## Supplementary Materials

The following are available online at https://www.mdpi.com/article/10.3390/microorganisms9102116/s1, Table S1: The 37 Serotypes Detected by the CDC’s *S. pneumoniae* qPCR Assay and Fluorescent Dye Associated with Each Probe, Table S2: Detection of Additional Serotypes by qPCR in Samples with Quellung Results Which Are Not Included in the 37-Serotype Molecular Assay, Table S3: Serotype Prevalence in Nasopharyngeal Samples Sent to NML, 2016 and 2018 combined. Samples had up to 4 serotypes Co-Carriage, All Serotypes are Counted.
