# Supplementary material for: PCR and Culture Analysis of Streptococcus pneumoniae Nasopharyngeal Carriage in Healthy Children
Source: Microorganisms. 2021 Oct 8;9(10):2116. doi: 10.3390/microorganisms9102116 (PMC8538797; doi:10.3390/microorganisms9102116)
Supplement: Supplementary file 1 [file microorganisms-09-02116-s001.zip › microorganisms-1403223-supplementary.pdf]

Table S1. The 37 Serotypes Detected by the CDC's *S. pneumoniae* qPCR Assay and Fluorescent Dye Associated with Each Probe.

Fluorescent Dye	Reaction 1	Reaction 2	Reaction 3	Reaction 4	Reaction 5	Reaction 6	Reaction 7
Fam	19A	6CD	15AF	1	6ABCD	14	5
Hex	3	22FA	33FA 37	16F	9VA	18CFBA	2
Cy5	7FA	12FAB 44 46	23A	11AD	4	19F	23F

Table S2. Detection of Additional Serotypes by qPCR in Samples with Quellung Results Which Are Not Included in the 37-Serotype Molecular Assay.

Year	Culture Serotype	PCR Serotypes [a]
2016	23B	11AD, 4
2016	15B	7FA
2016	35B	5, 15AF
2016	35B	3, 7FA, 22FA
2016	21	22FA, 5
2016	15B	12FAB 44 46
2016	10A	4
2016	21	12FAB 44 46
2016	19A	NT
2016	15B	5
2016	7C	1
2016	15B	16F, 4
2016	15B	23A
2016	23B	22FA
2018	35B	7FA
2018	23B	16F, 22FA
2018	35F	11AD
2018	15B	22FA
2018	35B	4
2018	21	4
2018	15B	4
2018	10A	5, 6AB
2018	15C	11AD
2018	35B	5
2018	23B	33FA_37
2018	21	5
2018	15B	15AF
2018	23B	22FA
2018	15B	15AF
2018	9N	4
2018	15C	15AF

[a] In the case of carriage by multiple serotypes, PCR serotypes/serogroups are separated by commas.

Table S3. Serotype Prevalence in Nasopharyngeal Samples Sent to NML, 2016 and 2018 combined. Samples Had Up to 4 Serotypes Co-carriage, All Serotypes are Counted.

		**Culture 2016 (*n* = 682)**	**PCR 2016 (*n* = 682)**	**Culture 2018 (*n* = 800)**	**PCR 2018 (*n* = 300)[a]**
Inclusion in Vaccines	Serotype	*n* (%)	*n* (%)	*n* (%)	*n* (%)
PCV7/PCV13	4	0 (0.0)	23 (3.4)	0 (0.0)	11 (3.7)
PCV7/PCV13	**6AB**CD [b]	6A: 1 (1.4)	4 (0.6)	0 (0.0)	15 (5.0)

PCV7/PCV13	**9V**A	0 (0.0)	1 (0.2)	0 (0.0)	3 (1.0)
PCV7/PCV13	14	0 (0.0)	0 (0)	0 (0.0)	0 (0)
PCV7/PCV13	**18C**FBA	0 (0.0)	2 (0.3)	0 (0.0)	1 (0.3)
PCV7/PCV13	19F	1 (1.4)	9 (1.3)	3 (4.4)	6 (2.0)
PCV7/PCV13	23F	0 (0.0)	2 (0.3)	0 (0.0)	0 (0)
PCV13	1	0 (0.0)	9 (1.3)	0 (0.0)	8 (2.7)
PCV13	3	0 (0.0)	13 (1.9)	0 (0.0)	5 (1.7)
PCV13	5	0 (0.0)	16 (2.3)	0 (0.0)	5 (1.7)
PCV13-related type	6CD	6C: 3 (4.2)	8 (1.2)	6C: 1 (1.5)	3 (1.0)
PCV13	7FA	0 (0.0)	7 (1.0)	0 (0.0)	3 (1.0)
PCV13	19A	1 (1.4)	1 (0.2)	0 (0.0)	0 (0)
PPV23	**11A**D	11A: 4 (5.6)	36 (5.3)	11A: 2(2.9)	12 (4.0)
PPV23	**12 F**AB 44 46	0 (0.0)	7 (1.0)	0 (0.0)	11 (3.7)
PPV23 (15B)	15BC	15B: 8 (11.3)<br>15C: 7 (9.9)	N/A	15B: 7 (10.3)<br>15C: 7 (10.3)	N/A
PPV23	**22F**A	22F: 8 (11.3)	34 (5.0)	22F: 2 (2.9)	14 (4.7)
PPV23	**33F**A 37	33F: 1 (1.4)	4 (0.6)	33F: 1 (1.5)	6 (2.0)
NVT	7C	1 (1.4)	N/A	0 (0.0)	N/A
NVT	9N	0 (0.0)	N/A	1 (1.5)	N/A
NVT	10A	3 (4.2)	N/A	2 (2.9)	N/A
NVT	15AF	15A: 2 (2.8)	18 (2.6)	15A: 5 (7.4)	20 (6.7)
NVT	16F	1 (1.4)	6 (0.9)	2 (2.9)	5 (1.7)
NVT	21	7 (9.9)	N/A	7 (10.3)	N/A
NVT	23A	2 (2.8)	13 (1.9)	9 (13.2)	16 (5.3)
NVT	23B	8 (11.3)	N/A	5 (7.4)	N/A
NVT	34	2 (2.8)	N/A	0 (0.0)	N/A
NVT	35B	7 (9.9)	N/A	7 (10.3)	N/A
NVT	35F	3 (4.2)	N/A	6 (8.8)	N/A
Non-typeable by Quellung	NT	1 (1.4)	N/A	0 (0.0)	N/A
Not-typed by qPCR	NT	N/A	187 (52.1)	N/A	94 (49.2)
Missing serotype	Missing	0 (0.0)	N/A	1 (1.5)	N/A

[a] 300/800 samples were tested from 2018, all positives and a random sample of negatives. Only results from 300 samples are included. [b] The serotype from a serogroup that is contained in the vaccine is noted in bold.
